# Large segmental defects in midshaft clavicle nonunion treated with autologous tricortical iliac crest bone graft

**DOI:** 10.1177/17585732211064815

**Published:** 2021-12-17

**Authors:** Fleur AE van der Burg, Thomas PA Baltes, Peter Kloen

**Affiliations:** 1 Department of Orthopaedic Surgery, 26066 Amsterdam UMC, University of Amsterdam, Amsterdam Movement Sciences, The Netherlands

**Keywords:** clavicle, fracture, non-union, bone graft

## Abstract

**Background:**

To evaluate the use of intercalary iliac crest bone graft in the treatment of clavicle nonunion with a large segmental bone defect (3–6 cm).

**Methods:**

This retrospective study evaluated patients with large segmental bone defects (3–6 cm) after clavicle nonunion, treated with open reposition internal fixation and iliac crest bone graft between February 2003 and March 2021. At follow-up the Disabilities of the Arm, Shoulder and Hand (DASH) questionnaire was administered. A literature search was performed to provide an overview of commonly used graft types per defect size.

**Results:**

We included five patients treated with open reposition internal fixation and iliac crest bone graft for clavicle nonunion with a median defect size of 3.3 cm (range 3–6 cm). Union was achieved in all five, and all pre-operative symptoms resolved. The median DASH score was 23 out of 100 (IQR 8–24). An extensive literature search revealed that there are no studies describing the use of an used iliac crest graft for defects larger than 3 cm. Instead, a vascularized graft was typically used to treat defects sizes between 2.5 and 8 cm.

**Discussion:**

An autologous non-vascularized iliac crest bone graft can be safely used and is reproducible to treat a midshaft clavicle non-union with a bone defect between 3 and 6 cm.

## Introduction

Nonunion of midshaft clavicula fractures occurs in about 0.1–15% of all clavicle fractures.^1^ Clavicle nonunion is associated with disabling symptoms, including pain, deformity, impaired shoulder function, and reduced strength.^2^ A recent study showed that a clavicle nonunion affects health-related quality of life with an impact exceeding that of a nonunion of the tibia, femur, or humerus.^3^ Substantial segmental defects of more than 1.4–2 cm are likely more symptomatic, as shortening is associated with impaired abduction, flexion, and internal rotation.^4,5^ In addition, a significantly shortened clavicle can be perceived as aesthetically displeasing. In these patients, clavicular length can be restored by combining open reduction and internal fixation (ORIF) with graft interposition.

Commonly used autologous grafts are iliac crest bone graft (ICBG), a free vascularized fibula bone graft or a free vascularized medial femoral condyle bone graft. It is common practice to use an ICBG to bridge bone defects between 2.5 and 3 cm.^6^ Vascularized grafts, which require microvascular reconstructive expertise, are reserved for length restoration in bone defects larger than 3 cm and cases where previous surgical intervention has failed.^7,8^ Currently, there is no consensus whether cases with bone defects larger than 3 cm can be treated with an ICBG.

This study aimed to investigate the outcome of using ICBG in the treatment of segmental defects larger than 3 cm in a series of patients with a midshaft clavicle nonunion. We hypothesized that in a clavicle nonunion with a segmental bone defect larger than 3 cm, reproducible results can be achieved by utilizing autologous tricortical ICBG.

## Methods

Between February 2003 and March 2021, a total of 110 midshaft clavicle fractures and clavicle nonunions were treated with ORIF by the senior author (a fellowship-trained orthopaedic trauma surgeon). Inclusion criteria for this retrospective study were a clavicle nonunion (defined as a non-healed fracture after nine months post-injury without radiographic progression for three months) with a segmental defect of at least 3 cm. Patients treated with techniques other than ORIF and segmental tricortical ICBG were excluded. Union was assessed using conventional imaging or a CT-scan. Patients were followed-up clinically with radiographs obtained at six weeks, 3, 6, 9, and 12 months or until union. The non-union scoring system (NUSS) was calculated for each patient.^9^ The NUSS is used to classify nonunion severity and has a maximum of 100 points. A high NUSS indicates the most severe nonunion. The Disabilities of the Arm, Shoulder and Hand (DASH) questionnaire was sent to the patient's home address, who then returned the filled-in questionnaire by mail or during a checkup.^10^ The DASH questionnaire has thirty items that describe the physical function and symptoms of the upper extremity. Each item has five response options. The scores for each item are summed with a total score ranging from zero to hundred. A score of zero indicates no disability, a score of hundred means severe disability. Ethics approval was waived by the Institutional Review Board (W21_314 # 21.349).

### Surgical technique

**Figure 1.**
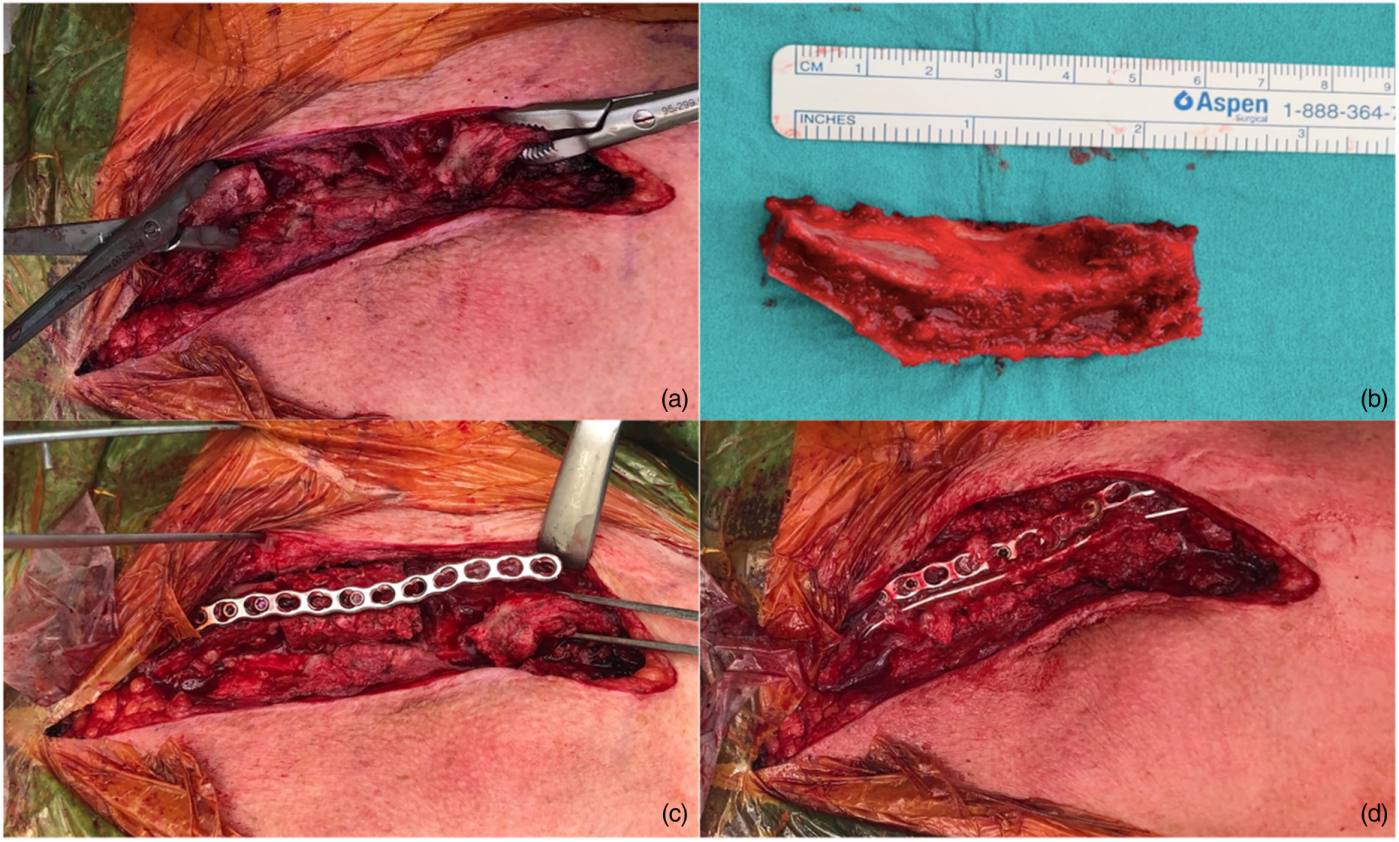
Intraoperative view. (a) View of the clavicular defect after the sclerotic cap has been removed; (b) Intercalary iliac crest bone graft of 5.5 cm; (c) Fixation of the intercalary iliac crest bone graft with a 2.7 mm locking-compression plate along the superior border of the clavicle; (d) After fixation of the superior plate an additional 2.7 mm LCP is placed on the anteroinferior border of the clavicula. In addition, cancellous bone and 5 cc demineralized bone matrix is added to the nonunion.

Based on a pre-operative CT, the required graft length is determined by measuring the contralateral healthy clavicle. The patient is positioned in a beach-chair orientation. The incision is parallel to the undersurface of the clavicle and centered over the nonunion. In case of a previous horizontal incision superior to the clavicle, we use the old incision as the skin, if mobile enough, still allows to position one of the plates anteroinferior. We do not try to identify and isolate the supraclavicular nerves. Although we are aware of the potential complication of a postoperative neuroma and/or dysesthesias in the region supplied by these small nerves, we have not encountered these problems in our patients so far. In midshaft nonunion, there is close proximity of the subclavian vein and artery and the brachial plexus to the undersurface of the bone. Adherence between the fibrous nonunion tissue or hypertrophic callus and the vessels can cause tearing of the vessel wall resulting in difficult-to-control bleeding. It is easier to work from lateral and medial aspects toward the nonunion, i.e., from healthy toward damaged tissue. All hardware is removed. At least five deep tissue cultures are taken. Intravenous antibiotic (2nd generation cephalosporin) is then given. Both nonunion ends are then freed from the surrounding scar tissue with a scalpel and/or rongueur. The ends of the nonunion often have a sclerotic cap that needs to be opened, reestablishing the medullary canal. With a 1.5- or 2.0-mm drill a few drill holes are made as deep as needed to observe blood coming out of the canal. This confirms adequate penetration through the, often sclerotic, end cap on either side of the nonunion (Figure 1(a)).

The tricortical iliac crest graft is then harvested, using a curvilinear incision over the iliac crest of about 8–10 cm. The graft is harvested with an oscillating saw or an osteotome. The graft is then shaped to fit into the defect, following the serpentine contour of the clavicle as good as possible (Figure 1(b)). To obtain good compression between graft and the nonunion we first fix the lateral side of the graft to the lateral end of the nonunion. We generally position a contoured 2.4-mm or 2.7-mm. recon LCP (DePuy Synthes, Amersfoort, The Netherlands) on the superior aspect to the lateral clavicle with at least two screws. We then used a pointed reduction clamp with one prong in the graft and one prong in a lateral plate hole and compress the graft to the lateral clavicle. We then place one locking screw through the plate into the graft (Figure 1(c)). Then we reduce the medial part of the nonunion to the medial end of the graft. Using the AO-tensioner device we compress the medial part to the nonunion to the medial aspect of the graft. The medial aspect to the plate is then fixated to the clavicle using cortical and/or locking screws. A 2.4- or 2.7-mm recon or straight LCP is then contoured and placed on the antero-inferior surface of the clavicle and secured into place with a combination of at least three screws placed on each side (Figure 1(d)). Using a sharp osteotome, approximately 1.5–2 cm on both sides of the nonunion are decorticated to create a bleeding bony surface for improved bone graft incorporation and stimulating local bone formation. Additional cancellous bone graft and/or demineralized bone matrix (DBX, DePuy Synthes, Amersfoort, The Netherlands) is added to both sides of the nonunion (Figure 1(d)). Patients are admitted overnight for pain control and then discharged on postoperative day 1 with follow-up 10–14 days postoperatively. Rehabilitation consists of pendulum exercises starting postoperative day 1. The patient is allowed to begin 10 kg weight bearing three weeks after surgery and if pain permits may begin full body weight bearing with exercises after three months if radiographs show signs of healing and the patient is pain-free. A double plating technique was applied as orthogonal plating using low-profile plates is hypothesized to increase resistance to multi-planar bending forces.^11^

### Literature search

On the 9th of January 2021 a PubMed search was conducted on the use of grafts in clavicle nonunion. The search contained the keywords: clavicle, nonunion, non-union, pseudarthrosis and bone graft. A total of forty-three articles were retrieved. Two authors screened full-text articles on defect size, graft size and the use of an autologous graft. Articles that described the use of allografts, failed to describe the use of interposition segmental grafts or reported on pathological fractures were excluded. Of the included articles an overview was made presenting patient demographics, type of graft used, defect size, fracture location, time to union and functional outcome scores.

### Statistical analysis

Data were analyzed in an SPSS database. For continuous data, it was first established whether the data had a normal distribution. The mean and standard deviation (SD) were used for data with a normal distribution. For data with a non-normal distribution, the median and interquartile range (IQR) were calculated. Prior, the distribution was determined by plotting a histogram, and the distribution was visually inspected. If the distribution was uncertain with a visual inspection, the distribution of the dependent variable was tested by using the Shapiro-Wilk test; if the result was non-statistically significant (above *p* = 0.05), the variable was normally distributed.

## Results

We identified five patients with a midshaft clavicle nonunion treated with internal fixation and length restoration using an autologous ICBG larger than 3 cm (Table 1). Two out of five patients were male. The median age at the time of surgery was 55 years (IQR 44–62). None of the patients had relevant co-morbidities, and none smoked. Only one patient had undergone radiation of the right chest in the past However, her nonunion concerned the contra-lateral left clavicle. The included patients had undergone a median of 3 (IQR 2–5) previous surgeries and had a median duration of nonunion of 5 years (IQR 4–44). Only one patient had no previous surgeries and was only treated conservatively. For the other four patients that were previously treated surgically, all available radiographs of the previous surgeries were inspected for possible reasons of failure. The most common reason for failure seemed to be using a bridging construct in a nonunion, with a lack of compression. Although this could not be quantitated (however this was discovered by discussing the operative technique with the referring physician), there seemed to have been a lack of “petalling” of the nonunion to refresh the fracture site and increase the contact area with the cancellous bone graft or DBX. Symptoms varied from pain, reduced range of motion, and globus sensation. The median NUSS was 21 (IQR 18–25). The median bone defect size was 3.3 cm (IQR 3–5 cm). The mean follow-up was 13 months (SD 3). Union was achieved in all five patients. Four patients regained full range of motion post-operatively. The median reported DASH score was 23 (IQR 8–24). In all patients, the pre-operative symptoms improved. One patient complained of persistent pain and functional impairment, which was possibly a result of hardware on the AC-joint. As it was premature to remove the entire plate, surgery was done to shorten the plate. Following this surgical procedure, the symptoms resolved. One patient was diagnosed with a possibly iatrogenic musculocutaneous traction injury, that caused slight hypoesthesia (15% deficit) of the lower left arm, hand and fingers.

**Table 1.** Patient characteristics.

Patiënt	Mechanism of injury	Age/Sex	Dominant arm	Occupation	Co-morbidities	Smoker	Pre-operative symptoms	NUSS	AO/OTA	Location	Fracture side
1	Handball	55/F	R	Administrative work	–	No	Pain	26	Unknown	Midshaft	L
2	Fall with bicycle	48/M	R	Bicycle mechanic	–	No	Reduced ROM and pain	21	15.2.C3	Midshaft	R
3	Unknown	39/F	R	N.A.	–	No	Pain and globus sensation	21	15.2.B2	Midshaft	L
4	Fall with bicycle	62/F	R	Teacher	–	No	Pain and globus sensation	24	15.2.B1	Midshaft	L
5	Fall with bicycle	61M	L	Administrative work	–	No	Pain	15	15.2.A2	Midshaft	L
Patiënt	Segmental defect (cm)	Iliac graft size (cm)	Duration of nonunion (yr)	Prior surgical interventions	Follow up (mo)	Time to union (mo)	DASH score	ROM	Post-operative symptoms	Complications/additional surgery	
1	6	6	34	3	18	6	23.3	Full	None	Musculocutaneous nerve traction injury with sensory loss forearm	
2	3	3	3.5	4	12	9	25	Abduction 100, Flexion 100	None	Hardware removal	
3	3.3	3.3	4	3	12	6	22.5	Full	None	None	
4	4.5	5.5	5	5	12	3	12	Abduction 130, Flexion 80	Pain	None	
5	3.2	3.2	54	0	12	3	4	Full	None	None	

NUSS: non-union scoring system; ROM: range of motion; DASH: Disabilities of the Arm; Shoulder and Hand; F: female, M: male; L: left; R: right; mo: months; mo: months; yr: years; N.A.: not available.

### Literature search

After full text screening of forty-three articles, seventeen articles were excluded because they described the use of allografts (*n* = 2), failed to describe the use of interposition segmental grafts (*n* = 8), reported on pathological fractures (*n* = 1) or the article was not available (*n* = 5). Of the twenty-six included articles, fourteen reported on the use of an ICBG (Table 2). In current literature ICBG is primarily used for midshaft clavicular defects smaller than 3 cm (Table 2). Ten articles reported the use of a vascularized segmental graft. In these studies, restored length ranged from 2.5 to 8 cm. No studies using an ICBG for a defect larger than 3 cm were identified.

**Table 2.** Literature overview on graft use in clavicle nonunion repair surgery.

Authors	*N* (age)	Graft	Defect size (cm)	Fixation	Nonunion location	Time to union (months)	Outcome score
Lim^12^	34 (M 40)	Iliac crest	1 ± 0.5	Plate	Midshaft	4	qDASH 6.1, VAS 1.3
Zhang^13^	12 (M 40)	Iliac crest	NA	Plate	Midshaft	2–2.5	SF-36 94.2, CS 77.1
Hollo^14^	25 (M 53)	Iliac crest	<3	Plate	Midshaft and lateral	NA	CS 82, SST score 12
Wiss^15^	3	Iliac crest	NA	Plate	Midshaft	NA	NA
Beirer^16^	17 (M 44)	Iliac crest	NA	Plate	Midshaft and lateral	NA	CS 82
Schnetzke^17^	58 (M 39)	Iliac crest	NA	Plate	Midshaft	NA	CS 82, DASH 21, SF-36 86
Kirchhoff^18^	10	Iliac crest	NA	Plate	Midshaft	NA	NA
O’Connor^19^	24 (M 40)	Iliac crest	NA	Plate	NA	NA	NA
Ballmer^20^	9	Iliac crest	NA	Plate	Midshaft	<6	NA
Ring^21^	2 (M 57)	Iliac crest	NA	Plate	Midshaft	NA	NA
Ebraheim^22^	16 (M 34)	Iliac crest	NA	Plate	NA	NA	NA
Olsen^23^	16 (M 34)	Iliac crest	NA	Plate	Midshaft	NA	NA
Jupiter^24^	3 (M 24)	Iliac crest	NA	Plate	Midshaft	NA	NA
Rollo^25^	42 (17–63)	Iliac crest <2 cm defect, Homologous graft >2 cm		Plate	Midshaft	3.5	DASH 16.6
Belyea^26^	1 (24)	Vasc graft (MFC)	2.5	Plate	Midshaft	NA	NA
Jaloux^27^	5 (M 38.4)	Vasc graft (MFC)	3.4 (2–5)	Plate	Midshaft	8.7	DASH 19.6
Arenas-Miquelez^28^	1 (50)	Vasc fibula	6	Plate	Lateral	1.5	CS 72, VAS 2
Huang^29^	7 (M 40)	Vasc graft (MFC)	2–5	Plate	Midshaft	4	VAS 1.6
Lenoir^30^	2 (M 51)	Vasc graft (MFC)	6	Plate	Midshaft	NA	CS 93
Abarca^31^	4 (M 55)	Vasc fibula	5–12	Plate	Midshaft	6	NA
Fuchs^32^	3 (M 40)	Vasc graft (MFC)	NA	Plate	Midshaft	5	NA
Erdmann^33^	2(M 47)	Vasc fibula	4–5	Plate	Midshaft	NA	NA
Momberger^34^	3 (M 35)	Vasc fibula	3, 5, 8	Plate	2 Midshaft, 1 Lateral		NA
Wood^35^	2	Vasc fibula	6	Plate	Midshaft	NA	NA
Calori^36^	1	Masquelet, 2 stage: RIA, MSC, BMP7	4	Plate	Midshaft	9	CS 90

M: mean; NA: not available; MFC: medial femoral condyle; Vasc: Vascularized; RIA: reamer irrigator aspirator; MSC: mesenchymal stem cells; BMP7: bone morphogenetic protein 7; CS: constant score; SF: 36-Item Short Form Survey; VAS: visual analogue scale; DASH: Disabilities of the Arm, Shoulder and Hand; qDASH: Quick Disabilities of the Arm, Shoulder and Hand; SST: simple shoulder test.

### Case description 1

**Figure 2.**
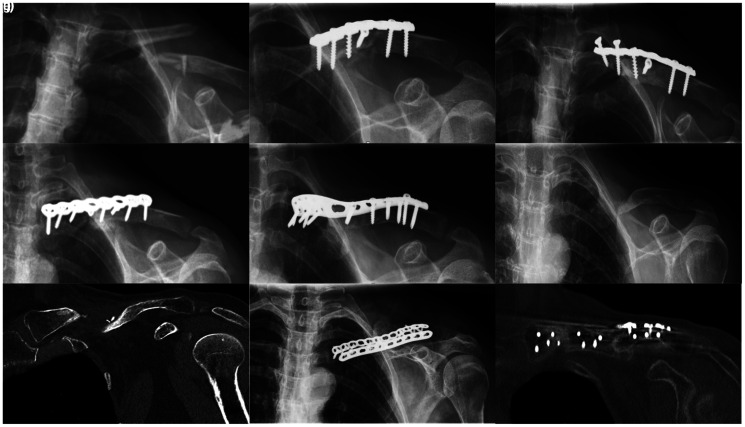
Imaging of left clavicle in chronological order. (a) Radiograph of the initial left midshaft clavicle fracture; (b) Radiograph of the initial osteosynthesis with a superior plate; (c) Radiograph of a failed osteosynthesis of the initial treatment, with loosening of the medial fixation, consistent with a nonunion: (d) Radiograph after first revision osteosynthesis with superior plate one and a half year post injury; (e) Radiograph, after plate osteosynthesis and ICBG interposition 3 years after initial surgery, which shows a single plate positioned on the superior aspect of the clavicle; (f and g) Radiograph and CT-scan 5 years post injury (after removal of osteosynthesis material) demonstrates shortening and slight angulation of the clavicle as a result of non-union; (h and i) Our post-operative radiograph and CT-scan 6 years post injury, after plate osteosynthesis and ICBG interposition, demonstrating dual-plating of the clavicle using 2.7 mm LCP plates on the superior and anterior border of the clavicle, resulting in a healed nonunion and restoration of clavicular length.

A previously healthy female teacher fractured her left midshaft clavicle by falling off a bicycle (Figure 2(a)). She was initially treated with plate osteosynthesis (Figure 2(b)). One-year post-injury loosening of the medial part of the plate and a nonunion of the clavicle resulted in revision plate osteosynthesis (Figure 2(c) and (d)). Two years after the initial injury the nonunion persisted and she underwent surgery for hardware removal. Intra-operative cultures grew staphylococcus epidermis which was treated with intravenous antibiotics. Three years post-injury she underwent repair with plate osteosynthesis and ICBG interposition (Figure 2(e)). One year after her third surgical intervention the plate was removed due to complaints of prominent hardware. On the pre-operative CT-scan, consolidation of the left clavicle midshaft was reported. This was allegedly confirmed by the operating surgeon when he removed the plate. Six months later, the patient complained of pain and a globus sensation in her throat. An MRI and plain radiographs revealed a nonunion (Figure 2(f) and (g)). The nonunion was then referred to us 5 years post-injury and treated with an ICBG and repeat plate fixation. Intraoperatively the defect size was around 4.5 cm (Figure 1(a)). A tricortical ICBG of 5.5 cm was placed with invagination of the native lateral clavicle into a cavity created in the IGBG (Figure 1(b)). Fixation was done with two plates (Figure 1(d)). Intra-operative cultures remained negative. Union was seen on a CT-scan three months after surgery (Figure 2(h) and (i)). At latest follow-up 12 months after surgery, the patient had had a full range of motion of the left shoulder and her pain had resolved completely. There were no complications at the surgery and donor site.

## Discussion

Our results show that large segmental defects (between 3 and 6 cm) can be successfully treated with a non-vascularized ICBG by applying the basic principles of nonunion treatment. These principles include, (1) debridement, removal of sclerotic bone ends, (2) alignment, restoration of length, (3) compression, and (4) rigid fixation. We believe that the use of 2 small, rather than one large plate increases the stability of the construct. It is well-known that rotational and bending forces are large on the clavicle. Orthogonal positioning of the plate with multiple locking and/or standard screws in the tricortical graft seem to provide enough stability during the ingrowth of the graft into both ends of the nonunion.

Currently it is common practice to treat clavicular bone defects larger than 3 cm with vascularized autografts. Several case series reporting the outcome of surgical treatment with vascularized autografts have been published.^26–35^ In the largest reported case series of seven patients, with segmental defects between 2 and 5 cm, consolidation was achieved in all patients using a vascularized medial femoral condyle autograft.^29^ Smaller series describing the clinical outcome of vascularized medial femoral condyle grafts and vascularized fibular grafts in segmental defects between 2 and 12 cm reported consolidation in 60–75% of the patients.^27,31^

However, a recent systematic review by Allsopp et al. found that compelling evidence for the use of vascularized bone grafts in large segmental defects is lacking.^37^ Vascularized grafts lead to prolonged operative time, an extended in-hospital stay and infection and bleeding are common complications.^38,39^ None of these complications were observed in the treatment of our patients with an ICBG. As good clinical results can be achieved with the use of ICBG the use of vascularized grafts might be considered obsolete in clavicle nonunion with large segmental defects.

Donor site morbidity should be analyzed when comparing the supplementary autologous bone graft used in nonunion repair surgery. Donor site complications following ICBG harvesting are not uncommon. Most common complications include gait disturbance (1.5%), painful scarring (9%), chronic pain (1.4%), and (mostly temporary) nerve deficit of the anterolateral femorocutaneus nerve.^40^ In contrast, when using a vascularized fibula graft temporary loss of peroneal sensory or motor function is a common finding.^41^ Other donor site complications associated with vascularized fibula graft harvesting are mild pain or discomfort, decreased ankle mobility and instability, and hammer or claw toe contracture.^42^

This is the first study describing the use of ICBG for the treatment of segmental defects between 3 and 6 cm. The study provides a clear description of the history, surgical technique and functional outcome. In all patients a postoperative CT scan was acquired to confirm osseous union and no patients were lost to follow-up. Despite the strength of this study, it has limitations inherently associated with its study design. The number of patients is relatively small as clavicle nonunion with a significant segmental defect (>3 cm) is rare. In addition, a pre-operative DASH score was not available. An established nonunion score (NUSS) was calculated for each patient. However, this score has mostly been used for nonunion of the femur and tibia. Given our relatively small group of patients it is unclear if the NUSS can be used as a measure for severity. Smoking and diabetes are weighted relatively high in the NUSS, whereas these are not clearly associated with a clavicle nonunion. Finally, the senior author has a tertiary nonunion referral practice with extensive experience in -recalcitrant- nonunion surgery. Results may therefore vary when this technique is applied by a less experienced surgeon.

In our cohort all 5 patients with a midshaft clavicle nonunion with a large segmental defect demonstrated radiographic union during a median follow up period of 13 months. All patients experienced improvement of symptoms. We conclude that using an ICBG to restore length in clavicle nonunion in defects up to 6 cm is a safe procedure with a high success rate.
